# Abdominal Pregnancy in the Small Intestine Presenting as Acute Massive Lower Gastrointestinal Hemorrhage

**DOI:** 10.1155/2017/8017937

**Published:** 2017-11-29

**Authors:** Apiradee Pichaichanlert, Vor Luvira, Nakhon Tipsunthonsak

**Affiliations:** ^1^Department of Surgery, Faculty of Medicine, Khon Kaen University, Khon Kaen, Thailand; ^2^Surgery Unit, Khon Kaen Hospital, Khon Kaen, Thailand

## Abstract

An abdominal pregnancy is an ectopic pregnancy in which the implantation site occurs in the abdominal cavity outside the female reproductive organs. There have been four reported cases that ruptured into the gastrointestinal tract and into the large intestine. We present the first case of an abdominal pregnancy rupturing into the small intestine with a good outcome.

## 1. Introduction

Acute lower gastrointestinal hemorrhage (LGIH) is defined as the onset of hematochezia, originating from either the colon or rectum. The most common causes of acute severe LGIH include diverticulosis, angioectasia, postpolypectomy bleeding, and ischemic colitis [1]. Ectopic pregnancy refers to the implantation of an embryo outside the uterus and is classified into two major types, according to the location of the implant—viz., a tubal or a nontubal pregnancy. The most common type of ectopic pregnancy (95%) is the tubal, which includes all parts of the fallopian tube (i.e., the fimbria, ampulla, isthmus, and cornual or interstitial part). An abdominal pregnancy is a nontubal pregnancy in which the implantation site occurs in the abdominal cavity and has a very low incidence (1%) [2]. Massive rectal bleeding is an unusual complication of ectopic pregnancy and carries with it a high mortality rate. We reviewed all cases of ectopic pregnancy that presented with LGIH [3–15] (Table 1) and only four of these were abdominal pregnancies.

For all reported cases of abdominal pregnancy presenting with lower GI bleeding, the gestational sac ruptured into the large intestine (colon or rectum). Herein, we present a case of an abdominal pregnancy in which the gestational sac ruptured into the small intestine and presented as severe hematochezia.

## 2. Case Presentation

A 32-year-old woman sought care at a provincial hospital after passing loose and dark stool about 10 times in a single day. She had been healthy until the diarrhea occurred and was not taking any medications. Her past medical history was unremarkable, except that she had undergone tubal surgery for pelvic inflammatory disease three years priorly. She had one child who was born by vaginal delivery. She had never undergone an instrumental pregnancy termination or intrauterine device insertion, which might lead to uterine perforation. She had no history of amenorrhea or abnormal vaginal discharge. Her initial diagnosis was upper gastrointestinal hemorrhage, but this was changed when bile was observed in the nasogastric tube after which the patient developed exsanguinating hematochezia and severe hypotension requiring 11 units of packed red cell transfusion for stabilization. Soon after, she was transferred to a tertiary care center.

**Figure 1:**
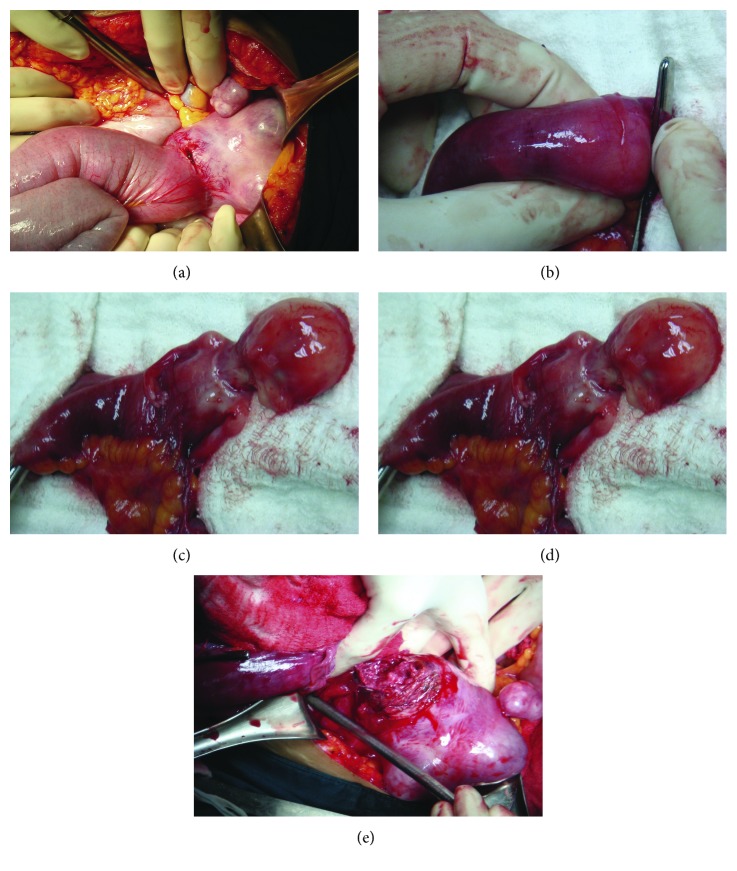
Photographs of intraoperative findings. (a) Exploratory laparotomy showing the adherence between the ileum and uterine fundus. (b) The intraluminal ileal mass palpable near the adherence site. (c) The opened ileum revealing a dead fetus. (d) Photograph showing the placenta in the uterus after detaching the ileum from the uterine fundus.

The patient's blood pressure was 70/40 mmHg, and her pulse rate was 120 beats/min. The abdominal examination was unremarkable. The per-rectal examination revealed excessive bleeding without any discernible cause. The laboratory tests showed a hemoglobin level of 6.3 g/dL and a platelet count of 28 × 10^3^/µL. Owing to the unstable condition of the patient, an emergency exploratory laparotomy was conducted in order to localize and control the bleeding. During the laparotomy, blood was found in the peritoneal cavity and a segment of the ileum attached to the fundus of uterus (Figure 1). The intraluminal content was palpated in the adhered ileal segment (Figure 1). An enterotomy revealed a fetus 7.5 cm in crown-rump length and fresh blood in the ileal lumen (Figure 1). The placental tissue had implanted at the fundal dome of the uterus and eroded into the small bowel (Figure 1). A segmental small bowel resection was performed along with reanastomosis. The placental tissue was removed by way of a wedge resection of the uterine wall. The patient had an uneventful postoperative course and was discharged on postoperative day 7.

**Figure 2:**
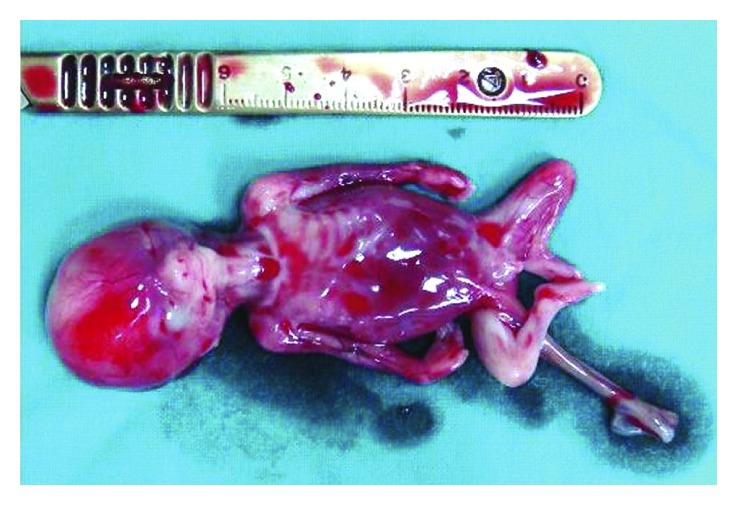
Photograph of the dead fetus.

Histologic examination of the resected specimens later confirmed the diagnosis of an abdominal pregnancy which included a male fetus of 4 months' gestational age (Figure 2), normal cord, subserous uterine myoma, and submucosal hemorrhage in the small intestine.

## 3. Discussion

A case of acute massive lower gastrointestinal hemorrhage, caused by an abdominal pregnancy that ruptured into the small intestine, is discussed. This is a rare gynecologic condition, considered to be an underlying cause of rectal bleeding, and is usually misdiagnosed, resulting in delayed treatment. Due to the rarity of the condition and the associated high mortality of abdominal pregnancy, despite having a high index of suspicion, it is not possible to diagnose the condition before surgery.

Lower gastrointestinal hemorrhage is defined as bleeding originating distal to the ligament of Treitz. Typically, massive bleeding is thought to require more than 3 to 5 units of blood transfused over 24 h. Although LGIH can occur at any age, the disease presentation for adults trends to be diverticular bleeding, inflammatory bowel disease, or neoplasm, and with advancing age, bleeding from an arteriovenous malformation, diverticular bleeding, or neoplasm [16]. The other common causes of LGIH are ischemic colitis, postpolypectomy bleeding, hemorrhoids, and stercoral ulcer.

We searched for reports on intestinal hemorrhage associated with ectopic pregnancy and found only 13 cases (Table 1). The site of the ectopic pregnancy was not identified in the first two reported cases. The first [3] was a case of a syphilitic woman six months pregnant, dying of hemorrhage and suddenly passing bloody stool containing fetal bones, and the second [4] was a case of rectal hemorrhage, proven at autopsy to have arisen from an ectopic gestational sac rupture into the sigmoid colon. The other 11 reports in the literature identified the implantation sites; for most of which (six cases) the implantation site was at the uterine cornu—that is, interstitial pregnancies, accounting for 1% of tubal pregnancies [13]. The literature indicated that the ectopic gestational sac usually ruptured into the ileum in cases of interstitial pregnancy. Four of the 13 cases were abdominal pregnancies, and the implantation site was usually situated at the uterine serosa and typically ruptured into the large intestine. The remaining case was a tubal ectopic pregnancy that ruptured into the sigmoid colon.

All types of ectopic pregnancies result in high maternal mortality, caused by delayed treatment of life-threatening hemorrhages. It is estimated that a woman with an abdominal pregnancy is 90 times more likely to die compared to a woman with an intrauterine pregnancy [17]. Historically, lack of knowledge of the condition was associated with mortality of the mother. Even today, the fetus almost always dies in ectopic pregnancies, although we did find one case of an abdominal pregnancy that presented as massive rectal bleeding, where both the mother and neonate survived [10].

The abnormal implantation site of an ectopic pregnancy leads to denigration of the trophoblastic tissue [13], which grows by invading the adjacent structures that are prone to the implantation site where there is greater blood supply. The communication of the intestinal tract with the ectopic gestational sac is called an enteroamniotic fistula, which follows the villous invasion of the bowel wall. When the gestational sac approximates to the intestine, there is an inflammatory reaction and an infection develops, resulting in a fistula. The vascularized gestational structures are aggravated by infection and villous invasion of the adjacent vascular structures, leading to massive hemorrhage [8]. The terminal ileum, sigmoid colon, and caecum are the parts of the gastrointestinal tract that are usually involved [14]. The small intestine is associated with interstitial and not abdominal pregnancies, which are usually associated with invasion of the large intestine. We have thus presented the first case of a ruptured abdominal pregnancy into the small intestine, which caused massive lower gastrointestinal hemorrhage.

Attending physicians need to be aware of the possibility of a fistula between the ectopic gestational sac and the bowel in any pregnant woman who presents with obscure intestinal hemorrhage, and to initiate prompt surgery. Although women with ectopic pregnancy frequently have no identifiable risk factors, a prospective case-controlled study revealed that increased awareness of ectopic pregnancy and knowledge of the associated risk factors help to identify women at higher risk and early diagnosis. In our case, we did not consider this condition initially but performed an emergency surgery after massive blood transfusions. During laparotomy—in order to control the bleeding and to prevent intraabdominal abscess formation or peritoneal sepsis—we resected the infected fistula, a segment of the small bowel, the wall of the involved uterus, and the ectopic fetus and placental tissue. After recovering from the surgery, the patient was discharged without any major morbidity.

In conclusion, abdominal pregnancy is a rare life-threatening condition requiring prompt treatment. This is the first report of an abdominal pregnancy that ruptured into the small intestine with a good outcome.

## Figures and Tables

**Table 1:** Reported cases of intestinal hemorrhage associated with ectopic pregnancy.

Author	Reported year	Patient	Site of ectopic pregnancy	Location of placental erosion	Gestational age (weeks)	Maternal outcome	Fetal outcome
Armstrong [3]	1835	NA	NA	NA	24	Dead	NA
Edgar [4]	1901	NA	NA	Sigmoid	NA	Dead	NA
Clark [5]	1932	Female, 25 years	Left interstitial	Sigmoid	6	Alive	Dead
Webster and Kerr [6]	1956	NA	Right interstitial	Appendix and ileum	NA	Dead	NA
Engel [7]	1961	NA	Left interstitial	Ileum	NA	Dead	NA
Shirkey et. al. [8]	1964	Female, 38 years	Left interstitial	Ileum	NA	Alive	Dead
Bigg et al. [9]	1965	Female, 19 years	Right interstitial	Caecum	NA	Alive	Dead
Bornman et al. [10]	1985	Female, 29 years	Abdominal (posterior wall of uterus)	Sigmoid	34	Alive	Alive
Seow et al. [11]	1992	NA	Abdominal	Caecum	NA	NA	NA
Verma et al. [12]	1996	Female, 38 years	Left fallopian tube	Sigmoid	4–8	Alive	Dead
Warshal et al. [13]	1996	Female, 36 years	Right interstitial	Ileum	12–16	Alive	Dead
Saravanane et al. [14]	1997	Female, 30 years	Abdominal (pouch of Douglas)	Rectum	14	Alive	Dead
Ekwaro et al. [15]	2004	Female, 26 years	Abdominal (fundus of uterus)	Sigmoid	NA	Dead	Dead
Present study	2017	Female, 32 years	Abdominal (fundus of uterus)	Ileum	16	Alive	Dead
